# Role of the α7 Nicotinic Acetylcholine Receptor in the Pathophysiology of Atherosclerosis

**DOI:** 10.3389/fphys.2020.621769

**Published:** 2020-12-23

**Authors:** Ildernandes Vieira-Alves, Leda M. C. Coimbra-Campos, Maria Sancho, Rafaela Fernandes da Silva, Steyner F. Cortes, Virgínia Soares Lemos

**Affiliations:** ^1^Department of Physiology and Biophysics, Institute of Biological Sciences (ICB), Universidade Federal de Minas Gerais, Belo Horizonte, Brazil; ^2^Department of Pharmacology, University of Vermont, Burlington, VT, United States; ^3^Department of Pharmacology, Institute of Biological Sciences (ICB), Universidade Federal de Minas Gerais, Belo Horizonte, Brazil

**Keywords:** vascular inflammation, atherosclerosis, α7nAChR, cholinergic signaling, cholinergic anti-inflammatory pathway

## Abstract

Atherosclerosis constitutes a major risk factor for cardiovascular diseases, the leading cause of morbidity and mortality worldwide. This slowly progressing, chronic inflammatory disorder of large- and medium-sized arteries involves complex recruitment of immune cells, lipid accumulation, and vascular structural remodeling. The α7 nicotinic acetylcholine receptor (α7nAChR) is expressed in several cell types involved in the genesis and progression of atherosclerosis, including macrophages, dendritic cells, T and B cells, vascular endothelial and smooth muscle cells (VSMCs). Recently, the α7nAChR has been described as an essential regulator of inflammation as this receptor mediates the inhibition of cytokine synthesis through the cholinergic anti-inflammatory pathway, a mechanism involved in the attenuation of atherosclerotic disease. Aside from the neuronal cholinergic control of inflammation, the non-neuronal cholinergic system similarly regulates the immune function. Acetylcholine released from T cells acts in an autocrine/paracrine fashion at the α7nAChR of various immune cells to modulate immune function. This mechanism additionally has potential implications in reducing atherosclerotic plaque formation. In contrast, the activation of α7nAChR is linked to the induction of angiogenesis and VSMC proliferation, which may contribute to the progression of atherosclerosis. Therefore, both atheroprotective and pro-atherogenic roles are attributed to the stimulation of α7nAChRs, and their role in the genesis and progression of atheromatous plaque is still under debate. This minireview highlights the current knowledge on the involvement of the α7nAChR in the pathophysiology of atherosclerosis.

## Introduction

Atherosclerosis cardiovascular disease (ASCVD) constitutes one of the leading causes of morbidity and mortality worldwide (Benjamin et al., 2017). Atherogenesis is a complex-multiphase pathology initiated by the progressive accumulation of low-density lipoprotein cholesterol (LDL-C) and other apolipoprotein B-containing lipoproteins in the subintimal space. These entrapped lipoproteins are exposed to local disturbed shear stress promoting endothelial dysfunction, which in turn leads to the synthesis of reactive oxygen species (ROS) by vascular endothelial, smooth muscle cells (VSMCs) and resident macrophages. Moreover, entrapped LDL particles become oxidized, generating oxidized LDL (oxLDL), and triggering sterile inflammation by upregulating the monocyte chemoattractant molecule-1 (MCP-1) and a variety of cell-adhesion molecules including intercellular adhesion molecule-1 (ICAM), P-selectin and vascular cell adhesion molecule-1 (VCAM-1) (Li et al., 1993). These molecules stimulate the adherence of circulating monocytes into the plaques. Infiltrated monocytes differentiate into distinct macrophage subtypes, which engulf LDL and produce several inflammatory mediators and cytokines. This new plaque milieu facilitates the migration of VSMCs from the media to the intima, where they further proliferate and shift to a less contractile and more secretory phenotype (Basatemur et al., 2019). Recruited macrophage and infiltrated leukocytes also undergo phenotypical switches to at least four classical phenotypes: M1, M2, M4, or Mmox (Tabas and Bornfeldt, 2016; Cochain and Zernecke, 2017; Cochain et al., 2018). This sequence of events is highly influenced by inflammatory mediators released by vascular cells and distinct subpopulations of innate/adaptive immune cells. Different cell types from the innate immune system were identified in mice and human plaques including mast cells, natural killers, dendritic cells (DCs), and neutrophils. Regarding the adaptive immune system, T and B cells are commonly found within atherosclerotic lesions. T cells are activated by LDL, presented by antigen-presenting macrophages and DCs. B-cell-derived plasma cells also produce serum antibodies against modified and oxLDL. B cells constitute a very heterogeneous population, comprising different functional subsets. Therefore, B-cells may play either atheroprotective or atherogenic roles, depending on the specific subset and their functionality (Sage et al., 2019). Altogether, experimental, clinical, and epidemiological research highlight the interplay between intraplaque immune cells and systemic inflammation as an important pathogenic mechanism in atherosclerosis.

**FIGURE 1:**
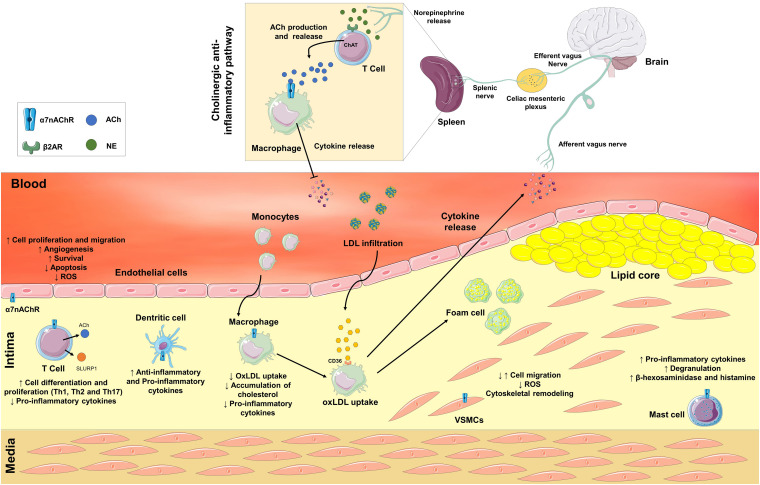
Neuronal and non-neuronal α7 nicotinic acetylcholine receptors (α7nAChR) are involved in the pathological process of atherosclerosis. In response to a variety of inflammatory stimuli, an afferent signal through the vagus nerve is triggered. Efferent vagal fibers are connected to the splenic nerve in the celiac mesenteric plexus. In the spleen, the splenic nerve releases norepinephrine, which activates β2 adrenergic receptors in T cells to release acetylcholine (ACh) that interacts with α7nAChR on macrophages and inhibits the release of pro-inflammatory cytokines. The Non-neuronal α7nAChR is also expressed in immune and vascular cells. Activation of α7nAChR in endothelial cells may stimulate angiogenesis, survival, apoptosis, increase cell proliferation and migration, and decrease the production of reactive oxygen species (ROS). In vascular smooth muscle cells (VSMCs), the activation of α7nAChR may promote both positive or negative modulation of cell migration, remodeling of the cytoskeleton, and a decrease in the production of ROS. In immune cells, the activation of α7nAChR may contribute to both anti- or proatherogenic processes depending on cell type. The activation of α7nAChR in T cells stimulates differentiation into Th1, Th2, and Th17 cells, and decreases the release of pro-inflammatory cytokines. Moreover, T cells synthesize and secrete ACh and Ly6/uPAR-related protein-1 (SLURP1). ACh and SLURP1 *via* paracrine signaling activate macrophages’ α7nAChR decreasing the release of pro-inflammatory cytokines, the uptake of oxidized LDL (OxLDL) and the accumulation of cholesterol inside macrophages. In dendritic and mast cells, ACh and SLURP1 increases the release of pro-inflammatory cytokines and degranulation, and the release of β-hexosaminidase and histamine in mast cells.

Atherosclerosis is a chronic disease in which inflammation is present during plaque initiation, progression, and even rupture. In each phase of the disease there are multiple inflammation-related pathways (Libby, 2012). The role of the autonomic nervous system in the regulation of inflammation has been extensively studied over the past decades (Borovikova et al., 2000; Pavlov and Tracey, 2005). In response to a variety of inflammatory stimuli, an afferent signal through the vagus nerve is triggered, activating efferent responses that attenuate tissue-specific cytokine production. This pathway, known as the “anti-inflammatory cholinergic reflex,” is mediated by the activation of the alpha-7 nicotinic acetylcholine (ACh) receptor (α7nAChR) in macrophages (Pavlov and Tracey, 2004), and linked to the genesis/development of atherosclerosis (Chen et al., 2016). In addition, the non-neuronal α7nAChR; expressed in vascular and immune cells; plays a crucial role in the pathology of atherosclerosis. In this minireview, we will highlight the complex role of α7nAChR in the pathogenesis of atherosclerosis (Figure 1).

## The α7-Nicotinic Acetylcholine Receptor

The ACh receptor (AChR) is a well-characterized membrane protein involved in the physiological responses to ACh in many neuronal and non-neuronal cells (Sharma and Vijayaraghavan, 2002; Dani and Bertrand, 2007; Fujii et al., 2008). These receptors are divided into two categories: (1) the muscarinic AChRs (mAChR), which belong to the superfamily of G protein-coupled receptors, is represented by five subtypes (M1–M5) (Hosey, 1992) and stimulated by muscarine. (2) The fast-ionotropic cationic nicotinic receptor channel (nAChR), activated by nicotine and involved in many pathophysiological disorders including Alzheimer’s and Parkinson’s disease, depression, and atherosclerosis.

Structurally, nAChRs constitute a large pentameric homo- or heteromeric assembly (290 kDa), which arises from the combination of 17 different subunits (α2–α10, β1–β4, γ, δ, and ε) with diverse pharmacological and physiological signatures (Sargent, 1993). Each subunit is composed of a relatively long extracellular N-terminal domain that contributes to ligand binding, 4 hydrophobic transmembrane domains (M1–M4), an intracellular loop between M3 and M4, and a short extracellular C-terminal end (Mckay et al., 2008).

Mammalian nAChRs are permeable to Na^+^, K^+^, and Ca^2+^, and adopt three principal transition states; (1) basal or resting (closed), (2) active (open), and (3) desensitized (closed) (Edelstein et al., 1996). The classic α7nAChR is the most abundant homologous nAChR subtype (5 α7 subunits) in the brain, where it was originally discovered and studied as a neuronal receptor. However, the α7nAChR is also expressed in a variety of non-neuronal cells, including T-cells, macrophages, vascular endothelium, VSMCs among others (Wang et al., 2003; Razani-boroujerdi et al., 2007; Li et al., 2010; Smedlund et al., 2011), where participates in the cholinergic anti-inflammatory pathway, angiogenesis (Wu et al., 2009), vascular remodeling and oxidative vascular stress (Li et al., 2014, 2018).

In neurons, the ligand-gated ion channel properties of α7nAChR have been extensively studied. This receptor exhibits high relative Ca^2+^ permeability (Seguela et al., 1993) and contains one extracellular ligand-binding site with high affinity for α-bungarotoxin (α-BTX) that rapidly and reversibly desensitize the receptor (De Jonge and Ulloa, 2007). However, very little is known about the channel properties of the non-neuronal α7nAChR.

## The Neuronal and Non-Neuronal Cholinergic System

The cholinergic system has an unquestionable role in neurotransmission as all its critical elements (choline acetyltransferase (ChAT), ACh, cholinesterase, and mAChRs and nAChRs) are present in the central/autonomic nervous system and at the neuromuscular junction (Brown, 2019).

The effects of ACh vary according to the predominance of receptor subtypes in the target tissue and until recently, they were reported to be mainly related to motor and cognitive processes (Picciotto et al., 2012). However, in 2000 Tracey and colleagues described the cholinergic system as a key element in the control of inflammation (Borovikova et al., 2000). The interplay between the neural pathway and immune cells to modulate inflammatory responses was termed “the inflammatory reflex.” In 2003, the same group described the α7nAChR as the target for the reduction in pro-inflammatory cytokines synthetized by macrophages and DCs (Wang et al., 2003). Interestingly, the splenic ACh discovered by Dale and Dudley (1929) is of non-neuronal origin as the spleen lacks cholinergic innervation. The physiological significance of this non-neuronal ACh remained undetermined until recent studies showing that spleenic cholinergic T cells (but not cholinergic neurons) constituted the source of ACh which stimulates α7nAChRs on splenic macrophages (Rosas-Ballina et al., 2011). Efferent vagal fibers are connected to the splenic nerve in the celiac mesenteric plexus. The splenic nerve releases norepinephrine, which stimulates β2 adrenergic receptors in T cells to further release ACh. ACh subsequently activates macrophage α7nAChRs, blocking the release of TNF, IL-1, HMGB1, and other cytokines (Rosas-Ballina et al., 2011). Since then, the role of the α7nAChR is considered essential in the pathophysiology of inflammatory diseases including rheumatoid arthritis (Maanen et al., 2010), sepsis (Ren et al., 2018) and atherosclerosis (Johansson et al., 2014).

These studies provide strong evidence that both neural and non-neuronal cholinergic systems effectively cooperate to control inflammation. In addition to immune cells, endothelial and VSMCs also possess the cholinergic signaling machinery (Wada et al., 2007; Pena et al., 2011; Fujii et al., 2017), being ACh involved in proliferation, differentiation, adhesion, migration, secretion, survival, and apoptosis *via* autocrine/paracrine pathways.

## The Non-Neuronal α7nAChR in the Regulation of Immune Cells

The recent discovery that lymphocytes (T and B), macrophages, and DCs synthesize ACh and express several types of mAChRs/nAChRs supports the existence of a local non-neuronal cholinergic system in immune cells (Reardon et al., 2013). In this regard, ACh secreted from CD4 + T cells stimulates α7nAChRs expressed by themselves or macrophages and DCs, decreasing the production of inflammatory cytokines (Fujii et al., 2017; Mashimo et al., 2019). Furthermore, activation of α7nAChRs on CD4 + T cells stimulates cell differentiation and proliferation (Treg cells and effector T cells) by antigen-dependent or independent pathways (Mashimo et al., 2019). Interestingly, T cells, CD205 + DCs, and macrophages express Ly6/uPAR-related protein-1 (SLURP-1) (Fujii et al., 2014), a positive allosteric ligand of the α7nAChR, which potentiates the effects of ACh (Chimienti et al., 2003). SLURP-1 has gained prominence in the cholinergic signaling of immune cells as it causes α7nAChR-dependent activation of T cells (Tjiu et al., 2011) and increases the production of ACh *via* enhancement of ChAT expression in human mononuclear cells and T cells (Fujii et al., 2014). Decreased production of TNF, IL-1beta, and IL-6 by human erythrocytes, T cells, and macrophages was also observed after activation of SLURP-1 (Chernyavsky et al., 2014).

The role of α7nAChR in distinct immune cells may differ depending on cell type and function. In macrophages, besides decreasing the release of inflammatory cytokines, α7nAChR stimulates the survival and polarization of the anti-inflammatory M2 phenotype (Lee and Vazquez, 2013). These findings support the notion that immune cells have their own cholinergic system. ACh and SLURP-1 modulate the cellular environment in an autocrine/paracrine way *via* α7nAChR expressed by DCs, macrophages, B and T cells, and culminating mostly in an anti-inflammatory profile.

## The Non-Neuronal α7nAChR in Endothelium and Smooth Muscle

The expression of the α7nAChR in the vasculature was initially described in bovine aortic endothelial cells (Conti-Fine et al., 2000). Shortly after, α7nAChRs were similarly identified in human endothelial cells from the microvasculature and umbilical veins, where they contribute to the angiogenic response to hypoxia and ischemia (Heeschen et al., 2002). Currently, it is well-established the modulatory role of the non-neuronal endothelial α7nAChR in both physiological and pathological angiogenesis (Cooke and Ghebremariam, 2008; Wu et al., 2009). Further studies described the activation of endothelial α7nAChR as an essential process in proliferation, migration, antioxidant, anti-inflammatory, senescence inhibition, and survival (Heeschen et al., 2002; Wu et al., 2009; Li et al., 2014, 2016; Liu et al., 2017). The underlying mechanisms of these effects involve a rise of intracellular Ca^2+^ concentration, activation of mitogen-activated protein kinase, phosphatidylinositol 3-kinase, endothelial nitric oxide synthase, and NF-κB, enhancement of Sirtuin 1 activity, and cyclin upregulation (Heeschen et al., 2002; Li and Wang, 2006; Wu et al., 2009; Li et al., 2014, 2016).

The α7nAChR is also expressed in VSMCs from rat aorta (Wada et al., 2007), guinea-pig basilar artery (Li et al., 2014), and human cerebral (Clifford et al., 2008) and umbilical arteries (Lips et al., 2005). In VSMCs, activation of α7nAChRs is associated with positive/negative modulation of migration, suppression of oxidative stress, inhibition of neointimal hyperplasia, abdominal aortic aneurysm, and cytoskeletal remodeling (Li et al., 2004, 2018, 2019; Wang et al., 2013; Liu et al., 2017). Interestingly, neovascularization, migration/proliferation of VSMCs, vascular remodeling, and oxidative stress contribute to plaque initiation and progression (Libby et al., 2019). Thus, the α7nAChR is considered as a unique element of the non-neuronal vascular cholinergic system, with a potential impact on the pathophysiology of atherosclerosis.

## Role of α7nAChRs in the Pathophysiology of Atherosclerosis

The cholinergic system, in particular the α7nAChR, has been widely linked to the pathophysiology of atherosclerosis (Santanam et al., 2012). The α7nAChR has been effectively identified in advanced atherosclerotic lesions of the human carotid artery (Johansson et al., 2014) suggesting its contribution to atherosclerosis. Numerous studies using murine models of atherosclerosis (summarized in Table 1) have either described an anti- (Hashimoto et al., 2014; Wang et al., 2017; Al-Sharea et al., 2017; Ulleryd et al., 2019) or pro-atherogenic role of the α7nAChR (Kooijman et al., 2015; Lee and Vazquez, 2015; Wang et al., 2017), being this aspect still an area of controversy in the literature.

**TABLE 1:** Involvement of the α7nAChR in the development of atherosclerosis in experimental models.

Model	Lesion induction time	Outcomes	References
Hematopoietic α7nAChR deficiency in LDLR^–/–^ mice	7 weeks	No differences in atherosclerotic lesion; ↑Leukocytes, monocytes, lymphocytes, and serum neutrophils.	Kooijman et al., 2015
Hematopoietic α7nAChR deficiency in LDLR^–/–^ mice	8 and 14 weeks	No differences in early atherosclerotic lesions. ↓Atherosclerotic lesion advance.	Lee and Vazquez, 2015
Hematopoietic α7nAChR deficiency in LDLR^–/–^ mice	8 weeks	↑Atherosclerotic lesion	Johansson et al., 2014
Total depletion (α7nAChR^–/–^)	No lesion	↑Cholesterol accumulation in macrophages; ↑Ox-LDL uptake by macrophages; ↓Macrophage cellular paraoxonase activity and gene expression.	Wilund et al., 2009
**Pharmacological α7nAChR agonists**			
GTS-21 in ApoE^–/–^ mice	8 weeks	↓Atherosclerotic lesion; ↓Lipid accumulation within the lesion; ↓Macrophage accumulation within the lesion; ↓Circulating monocytes.	Al-Sharea et al., 2017
AR-R17779 ApoE^–/–^ mice	4 weeks	↓Atherosclerotic lesion; ↓Gene expression of IL-1β, TNF-α, IL-6, NOX2 in the abdominal aorta; Survival rate.	Hashimoto et al., 2014
PNU-282927 ApoE^–/–^ mice	4 weeks	↓Atherosclerotic lesion; ↓IL-6 and serum TNF-α.	Chen et al., 2016
AZ6983 ApoE^–/–^ mice	8 weeks	↓Atherosclerotic lesion; ↓Lipid accumulation within the lesion; ↓Macrophage accumulation within the lesion.	Ulleryd et al., 2019
Varenicline ApoE^–/–^ mice	8 weeks	↑Atherosclerotic lesion.	Koga et al., 2014
Nicotine (α-bungarotoxin sensitive) in ApoE^–/–^KitW-sh/W-sh mice*	12 weeks	↑Atherosclerotic lesion; ↑Lipid accumulation within the lesion; ↑MCP-1, IFN-y, and TNF-α, IL-6 production by peritoneal macrophages.	Wang et al., 2017

*ApoE^–/–^ Kit^*W–sh/W–sh*^, mast cell-deficient mouse.

The hematopoietic deficiency of α7nAChR was evaluated with the aid of low-density lipoprotein receptor knockout mice (LDLR^–/–^), raising controversial findings. While Johansson et al. (2014) reported an acceleration of the development of atherosclerosis in high-fat diet fed mice (HFD; 8 weeks), Kooijman et al. (2015) showed no changes in atheromatous plaque formation.

Lee and Vazquez (2015) compared the impact of the α7nAChR hematopoietic deficiency between early and advanced atherosclerotic lesions (14 weeks of HFD) in LDLR^–/–^ mice. In the early stages, no significant changes in the development of atherosclerosis were observed, whereas in advanced lesions the lack of α7nAChRs resulted in the reduction of the lesion size, macrophage content, and cell proliferation, indicating a pro-atherogenic effect of the α7nAChR. Therefore, these results are quite controversial, and the underlying rationale is still unclear. However, it is remarkable that α7nAChRs are expressed in immune, endothelial and VSMC cells, and participate in multiple anti- and pro-atherogenic processes, which surely bring more complexity for the understanding of the role of this receptor in the pathophysiology of atherosclerosis.

Total depletion of α7nAChRs and its impact on atherosclerosis development was also tested. Macrophages from α7nAChR^–/–^ mice exhibited an increase in the uptake of oxLDL and cholesterol accumulation, as well as a decrease in macrophage’s antioxidant capacity *via* reduction of cellular paraoxonase expression (Wilund et al., 2009). These findings collectively support an anti-atherogenic effect mediated by the α7nAChR in macrophages.

The recruitment of immune cells to the lesion site and the release of inflammatory cytokines into the circulation are mechanisms involved in the progression of atherosclerosis. The activation of the anti-inflammatory cholinergic reflex is essential to decrease the production of TNF-α in the spleen (Rosas-Ballina et al., 2011), a crucial monocyte-producing organ for the development of atherosclerosis (Robbins et al., 2015). Interestingly, Chen et al. (2016) demonstrated that baroreflex dysfunction exacerbated atherosclerosis, and the activation of α7nAChRs with a selective agonist (PNU-282927) attenuated the development of atherosclerosis and decreased the size of the lesion in ApoE^–/–^ mice. Moreover, splenectomized ApoE^–/–^ mice displayed augmented atherosclerotic plaque size (Rezende et al., 2011). These data are in line with an anti-atherogenic role of immune cells’ α7nAChR.

As discussed above, the non-neuronal cholinergic system may modulate the development of atherosclerotic lesions. Accordingly, α7nAChR^–/–^ mice exhibited enhanced levels of circulating pro-inflammatory cytokines in plasma (Wilund et al., 2009) and carotid arteries (Li et al., 2018). Additionally, selective pharmacological activation of α7nAChRs decreased circulating monocytes, plasma pro-inflammatory cytokines, and the infiltration of inflammatory cells in atherosclerotic lesions (Hashimoto et al., 2014; Al-Sharea et al., 2017; Ulleryd et al., 2019). These results confirm that α7nAChR activation diminishes systemic inflammation and modifies the inflammatory phenotype of the plaque, consistent with an anti-atherogenic profile for α7nAChR.

During the development of atherosclerosis, macrophage-induced apoptosis is critical in the progression of the lesion. A recent study using bone marrow-derived macrophages (BMDMs) showed that AZ6983; a selective α7nAChR agonist; enhanced macrophage phagocytosis of apoptotic cells (Ulleryd et al., 2019). *In vivo* treatment with AZ6983 decreased the expression of CD47, a marker known to emit “don’t eat me” signals (Oldenborg et al., 2012) in the atherosclerotic lesion (Ulleryd et al., 2019). These studies are in line with an anti-atherogenic role for macrophages’ α7nAChR. Conversely, the activation of mast cells’ α7nAChR displays a pro-atherogenic profile (Wang et al., 2017). The increased number of mast cells was correlated to the progression of atherosclerosis in human coronary arteries, and to the progression and destabilization of the plaque in animal models (Kovanen et al., 1995; Bot et al., 2014). Therefore, the activation of α7nAChRs in different immune cell types may contribute to the controversial role of this receptor in atherosclerosis.

Pharmacological tools were also employed to study the contribution of α7nAChR in the development of atherosclerosis. Using bone marrow mononuclear cells (BMMCs) from ApoE^–/–^ mice, Wang et al. (2017) observed that nicotine treatment (100 μg/mL) activated mast cells, causing cell degranulation and β-hexosaminidase and histamine release. This effect was attenuated by mecamylamine or α-BTX, a non-selective and a selective antagonist of α7nAChRs, respectively. Interestingly, the supernatant of BMMCs (pre-treated with nicotine), increased pro-inflammatory cytokines MCP-1, IFN-y, TNF-α, and IL-6 by peritoneal macrophages. In human DCs, nicotine (0.1 μmol/L) enhanced the synthesis of pro-inflammatory IL-12 and anti-inflammatory IL-10 cytokines (Aicher et al., 2003), being the above effects blocked by α-BTX. Increasing evidence has demonstrated that nicotine increases atherosclerosis in ApoE^–/–^ mice through the activation of α7nAChRs in mast cells, supporting its pro-inflammatory effects (Wang et al., 2017). In contrast, pharmacological treatment of ApoE^–/–^ mice with the α7nAChR selective agonists PNU-282927 (Chen et al., 2016), AZ6983 (Ulleryd et al., 2019), 3-(2,4-dimethoxybenzylidene) anabaseine (GTS-21) (Al-Sharea et al., 2017), and AR-R17779 (Hashimoto et al., 2014), or acetylcholinesterase inhibitors (Inanaga et al., 2010) diminished atherosclerotic lesions and lipid accumulation within plaques. Therefore, pharmacological selectivity for the α7nAChR is crucial for an anti-atherogenic effect. Notably, both α1 (Zhang et al., 2011) and α3 nAChRs (Yang et al., 2016) were reported to modulate atherosclerotic plaque progression. Another critical characteristic of the α7nAChR is its rapid desensitization (Edelstein et al., 1996). Thus, high concentrations of α7nAChR ligands and long-term treatments may represent a bias for some studies.

## Conclusion

The involvement of the α7nAChR in the development of atherosclerosis is yet an expanding field. *In vivo* studies revealed both anti- or pro-atherogenic effects. *In vitro* studies indicated that the stimulation of α7nAChRs regulates the function of different cells involved in a diversity of pathways linked to plaque progression. Stimulation of vascular α7nAChRs contribute to angiogenesis and proliferation of VSMCs and may promote atherogenesis. In immune cells, α7nAChRs seem to exert anti- and/or pro-atherogenic effects depending on the cell type. In macrophages, α7nAChR stimulation causes atheroprotective effects as it prevents the synthesis of pro-inflammatory cytokines and chemotaxis, reduces lipid uptake, and improves the phagocytosis capacity of apoptotic cells. In dendritic and mast cells, α7nAChR stimulation causes destabilization and progression of atherosclerosis, increasing vascular inflammation. Due to all these effects, the α7nAChR represents a key element in the complex pathophysiology of atherosclerosis and a promising target for the treatment of vascular inflammation and atherosclerosis. Finally, the use of cell-specific α7nAChR knockout models, the development of highly selective α7nAChR agonists/antagonists, and a correct functional analysis on the contribution of the different nAChRs subtypes may aid in advancing our current knowledge on the impact of α7nAChRs in the pathophysiology of atherosclerosis.

## Author Contributions

IV-A, LC, MS, RS, SC, and VL wrote and revised the manuscript. All authors contributed to the article and approved the submitted version.

## Conflict of Interest

The authors declare that the research was conducted in the absence of any commercial or financial relationships that could be construed as a potential conflict of interest.

## Abbreviations

α 7nAChR: α 7 nicotinic acetylcholine receptor
α-BTX: α-bungarotoxin
ACh: acetylcholine
ASCVD: atherosclerosis cardiovascular disease
BMDMs: bone marrow-derived macrophages
ChAT: choline acetyltransferase
DCs: dendritic cells
LDLR^–/–^: low-density lipoprotein receptor depletion
oxLDL: oxidized low-density Lipoprotein
ROS: reactive oxygen species
SLURP-1: Ly6/uPAR-related protein-1
VSMCs: vascular smooth muscle cells.
